# Deliberate self-harm and suicide in people with immigrant background: how can reason for immigration and country of origin differentiate the risks?

**DOI:** 10.1192/j.eurpsy.2024.168

**Published:** 2024-08-27

**Authors:** P. Qin

**Affiliations:** National Centre for Suicide Research and Prevention, University of Oslo, Oslo, Norway

## Abstract

**Introduction:**

A growing body of research have devoted into suicide and deliberate self-harm in immigrant population, but no study has examined how reason for immigrating to the host country differentiates the risks.

**Objectives:**

To gain firm insight into suicide and deliberate self-harm among peopel with immigrant background.

**Methods:**

Norwegian registers were interlinked to identify all individuals who died by suicide in 1992-2018 and who received emergency treatment for non-fatal deliberate self-harm (DSH) in 2008-2018, and to construct the respective databases via a nested case-control design. Rates and relative risks of suicide and DSH were assessed according to immigrant background, country of birth and reasons of immigration, and in the context of personal socioeconomic status.

**Results:**

People with an immigrant background accounted for 11.6% of all suicides in 1992-2018 and 17.9% of all DSH incidents treated in hospital emergency departments in 2008-2018. The rates of both suicide and DSH were highest in people born abroad with two Norway-born parents (mean rate: 19.4/100 000 for suicide and 280.9/100 000 for DSH) and lowest in the second-generation immigrants. Compared with the native Norwegians, suicide risk was significantly higher for those foreign-born with two Norway-born parents (HR=1.50) and those born in Norway with 1 one foreign-born parent (HR=1.20), but was significantly lower for the first- and second-generation immigrants. The associated risks remained almost unchanged when the data were adjusted for personal differences in education, marital status, income and place of residence in Norway. The analyses on deliberate self-harm exhibited similar patterns of results as for suicide, although the estimated reduced risks in the first- and second-generation immigrant is somewhat smaller. Evidently, the risks for suicide and DSH varied significantly by reason of immigration and country of origin. Immigrants coming for education had the lowest risk for suicide and self-harm, and those coming for work the second lowest. The risks for immigrants coming for family unity were lower than the natives, but significantly higher than counterparts coming for job or education from the same country. Among immigrants coming to Norway as a refugee or asylum seeker, the risk of suicide was comparably high as those coming for work, but the relative risk for self-harm was significantly higher. The increased risks associated with the mixed immigration background tended to be slightly higher in females than in males, and were likely confined to adoptee population.

**Conclusions:**

Risks for suicide and deliberate self-harm in people with an immigrant background differs significantly by reason of immigration and country of origin. The findings should be taken into account in efforts of mental healthcare and suicide prevention targeting immigrant population.

**Disclosure of Interest:**

None Declared

